# Supplementary material for: Synthetic and genomic regulatory elements reveal aspects of cis-regulatory grammar in mouse embryonic stem cells
Source: eLife. 2020 Feb 11;9:e41279. doi: 10.7554/eLife.41279 (PMC7077988; doi:10.7554/eLife.41279)
Supplement: Transparent reporting form [file elife-41279-transrepform.docx]

***eLife’s* transparent reporting form**

We encourage authors to provide detailed information *within their submission* to facilitate the interpretation and replication of experiments. Authors can upload supporting documentation to indicate the use of appropriate reporting guidelines for health-related research (see [EQUATOR Network](http://www.equator-network.org/%20)), life science research (see the [BioSharing Information Resource](https://biosharing.org/)), or the [ARRIVE guidelines](http://www.plosbiology.org/article/info:doi/10.1371/journal.pbio.1000412) for reporting work involving animal research. Where applicable, authors should refer to any relevant reporting standards documents in this form.

If you have any questions, please consult our Journal Policies and/or contact us: [editorial@elifesciences.org](mailto:editorial@elifesciences.org).

**Sample-size estimation**

- You should state whether an appropriate sample size was computed when the study was being designed
- You should state the statistical method of sample size computation and any required assumptions
- If no explicit power analysis was used, you should describe how you decided what sample (replicate) size (number) to use

Please outline where this information can be found within the submission (e.g., sections or figure legends), or explain why this information doesn’t apply to your submission:

Sample-size estimation (number of barcodes per tested element and number of replicates) was based on previous empirical results (Fiore and Cohen, 2016) and therefore is not applicable to this submission.

**Replicates**

- You should report how often each experiment was performed
- You should include a definition of biological versus technical replication
- The data obtained should be provided and sufficient information should be provided to indicate the number of independent biological and/or technical replicates
- If you encountered any outliers, you should describe how these were handled
- Criteria for exclusion/inclusion of data should be clearly stated
- High-throughput sequence data should be uploaded before submission, with a private link for reviewers provided (these are available from both GEO and ArrayExpress)

Please outline where this information can be found within the submission (e.g., sections or figure legends), or explain why this information doesn’t apply to your submission:

Number of biological replicates performed is reported in the Methods section under header, ‘Cell culture and transfection’. Criteria for exclusion of barcodes with insufficient reads in the RNA or DNA pool are included in the Methods section under header, ‘Massively Parallel Reporter Assay’. Sequencing data for each replicate has been uploaded to GEO and a private link for reviewers was provided in our full submission email. Additionally, normalized data for each replicate has been included as a supplemental file.

**Statistical reporting**

- Statistical analysis methods should be described and justified
- Raw data should be presented in figures whenever informative to do so (typically when N per group is less than 10)
- For each experiment, you should identify the statistical tests used, exact values of N, definitions of center, methods of multiple test correction, and dispersion and precision measures (e.g., mean, median, SD, SEM, confidence intervals; and, for the major substantive results, a measure of effect size (e.g., Pearson's r, Cohen's d)
- Report exact p-values wherever possible alongside the summary statistics and 95% confidence intervals. These should be reported for all key questions and not only when the p-value is less than 0.05.

Please outline where this information can be found within the submission (e.g., sections or figure legends), or explain why this information doesn’t apply to your submission:

Description and justification of statistical analysis methods can be in Methods section under header ‘Other analysis and data sources’. Test choices, exact values of N, methods of test correction are reported in body of manuscript where figure showing data are referenced. Exact p-values are reported along with statistical tests with the exception of comparisons between barcodes for library elements/sequences and basal constructs where exact p-values are included in Sup. File 1C and Sup. File 1H.

(For large datasets, or papers with a very large number of statistical tests, you may upload a single table file with tests, Ns, etc., with reference to sections in the manuscript.)

**Group allocation**

- Indicate how samples were allocated into experimental groups (in the case of clinical studies, please specify allocation to treatment method); if randomization was used, please also state if restricted randomization was applied
- Indicate if masking was used during group allocation, data collection and/or data analysis

Please outline where this information can be found within the submission (e.g., sections or figure legends), or explain why this information doesn’t apply to your submission:

Samples were not allocated into experimental groups and therefore is not applicable to this submission.

**Additional data files (“source data”)**

- We encourage you to upload relevant additional data files, such as numerical data that are represented as a graph in a figure, or as a summary table
- Where provided, these should be in the most useful format, and they can be uploaded as “Source data” files linked to a main figure or table
- Include model definition files including the full list of parameters used
- Include code used for data analysis (e.g., R, MatLab)
- Avoid stating that data files are “available upon request”

Please indicate the figures or tables for which source data files have been provided:

For figures that present expression data for the Synthetic library (Figures 1A & 2), these data are included in Sup. File 1C: SYN_ExpressionSummary. For Figure 1B, these data are included in Sup. File 1H: GEN_ExpressionSummary. For Figure 3, these data are included in Sup. File 2E: SYN_FeaturesiRF and a full list of parameter terms are included in Sup. File 2A. For Figure 4, the data for panel A is included in Sup. File 2G: gkmSVM_8merOutputScores and panels B-H are included in Sup. File 2F: gWT_FeaturesiRF. For Figure 5 and 6, these data are included in Sup. File 2F gWT_FeaturesiRF and a full list of parameter terms are included in Sup. File 2B.
